# Supplementary material for: Inverted Gabor holography principle for tailoring arbitrary shaped three-dimensional beams
Source: Sci Rep. 2016 May 20;6:26312. doi: 10.1038/srep26312 (PMC4873839; doi:10.1038/srep26312)
Supplement: Supplementary Information [file srep26312-s1.doc]

**Inverted Gabor holography principle for tailoring arbitrary shaped three-dimensional beams**

T. Latychevskaia^1^* and Hans-Werner Fink

**Supplementary Note**

**MATLAB main code:**

% This code simulates diffractor which under illumination with a plane wave

% creates a light beam curves in a shape of cosine

% The code is written by Tatiana Latychevskaia, 2016

% Citation for this algorithm or any of its subroutines:

% Tatiana Latychevskaia and Hans-Werner Fink

% " Bending light on demand: Creating arbitrary three-dimensional shape and

% modulation of light by employing inverted hologram of arranged point-like

% absorbers" Scientific Reports NNN, 2016

%%%%%%%%%%%%%%%%%%%%%%%%%%%%%%%%%%%%%%%%%%%%%%%%%%%%%%%%%%%%%%%%%%%%%%%%%%

%%%%%%%%%%%%%%%%%%%%%%%%%%%%%%%%%%%%%%%%%%%%%%%%%%%%%%%%%%%%%%%%%%%%%%%%%%

%%%%%%%%%%%%%%%%%%%%%%%%%%%%%%%%%%%%%%%%%%%%%%%%%%%%%%%%%%%%%%%%%%%%%%%%%%

close all

clear all

% Input parameters to create cosine curve

N = 200; % number of steps in z directions

Nt = 2000; % steps to create a smooth curve, Nt > N

R = 80; % amplitude of the cosine function in pixels

P = 100; % period of the cosine function in pixels

% Input parameters to calculate diffractor

NH = 832; % diffractor size in pixels NH x NH

pixel = 32*10^(-6); % pixel size in meters

lambda = 650*10^(-9); % wavelength

dz = 0.004; % z-distance step between the object planes

z0 = 0.1; % z distance from the diffraction at which the curve starts

%%%%%%%%%%%%%%%%%%%%%%%%%%%%%%%%%%%%%%%%%%%%%%%%%%%%%%%%%%%%%%%%%%%%%%%%%%

%%%%%%%%%%%%% FORMATION OF 3d-COSINE CURVE %%%%%%%%%%%%%%%%%%%%%%

%%%%%%%%%%%%%%%%%%%%%%%%%%%%%%%%%%%%%%%%%%%%%%%%%%%%%%%%%%%%%%%%%%%%%%%%%%

deltat = (N-1)/(Nt-1);

object0 = zeros(N,N,N);

for ii = 1:Nt

t = deltat*(ii-1)+1;

x = round(N/2+1 + R*sin(2*pi*t/P));

y = round(N/2+1);

z = round(t);

object0(x,y,z) = 1;

end

%%%%%%%%%%%%%%%%%%%%%%%%%%%%%%%%%%%%%%%%%%%%%%%%%%%%%%%%%%%%%%%%%%%%%%%%%%

% object projection in (x,y)-plane

object0_xy = zeros(N,N);

for ii = 1:N

for jj = 1:N

for kk = 1:N

object0_xy(ii,jj) = object0_xy(ii,jj) + object0(ii,jj,kk);

end

end

end

figure

imshow(rot90(object0_xy(:,:)),[]);

%%%%%%%%%%%%%%%%%%%%%%%%%%%%%%%%%%%%%%%%%%%%%%%%%%%%%%%%%%%%%%%%%%%%%%%%%%

% object projection in (x,z)-plane

object0_xz = zeros(N,N);

for ii = 1:N

for jj = 1:N

for kk = 1:N

object0_xz(ii,jj) = object0_xz(ii,jj) + object0(ii,kk,jj);

end

end

end

figure

imshow(rot90(object0_xz(:,:)),[]);

%%%%%%%%%%%%%%%%%%%%%%%%%%%%%%%%%%%%%%%%%%%%%%%%%%%%%%%%%%%%%%%%%%%%%%%%%%

% object projection in (y,z)-plane

object0_yz = zeros(N,N);

for ii = 1:N

for jj = 1:N

for kk = 1:N

object0_yz(ii,jj) = object0_yz(ii,jj) + object0(kk,ii,jj);

end

end

end

figure

imshow(rot90(object0_yz(:,:)),[]);

%%%%%%%%%%%%%%%%%%%%%%%%%%%%%%%%%%%%%%%%%%%%%%%%%%%%%%%%%%%%%%%%%%%%%%%%%%

%%%%%%%%%%%%%%%%% ZERO-PADDING THE CURVE %%%%%%%%%%%%%%%%%%%%%%

%%%%%%%%%%%%%%%%%%%%%%%%%%%%%%%%%%%%%%%%%%%%%%%%%%%%%%%%%%%%%%%%%%%%%%%%%%

object = zeros(NH,NH,N);

for kk = 1:N

for ii = 1:N

for jj = 1:N

object(ii + (NH - N)/2, jj + (NH - N)/2, kk) = object0(ii,jj,kk);

end

end

end

%%%%%%%%%%%%%%%%%%%%%%%%%%%%%%%%%%%%%%%%%%%%%%%%%%%%%%%%%%%%%%%%%%%%%%%%%%

%%%%%%%%%%%%% HOLOGRAM SIMULATION %%%%%%%%%%%%%%%%%%%%%%

%%%%%%%%%%%%%%%%%%%%%%%%%%%%%%%%%%%%%%%%%%%%%%%%%%%%%%%%%%%%%%%%%%%%%%%%%%

area = NH*pixel; % area size in meters

transmission = 1 - object; % 3d transmission function of the object

wavefront = ones(NH, NH); % complex-valued wavefront

prop = propagator(NH, lambda, area, dz);

f = exp(-i*2*pi*dz/lambda);

for kk = 1:N

z_plane = kk

for ii = 1:NH

for jj = 1:NH

wavefront(ii,jj) = wavefront(ii,jj)*transmission(ii,jj,kk);

end

end

wavefront = f*FT2Dbacktat(FT2Dtat(wavefront).*prop);

end

prop0 = propagator(NH, lambda, area, z0);

f0 = exp(-i*2*pi*z0/lambda);

wavefront = f0*FT2Dbacktat(FT2Dtat(wavefront).*prop0);

hologram = abs(wavefront).^2;

%%%%%%%%%%%%%%%%%%%%%%%%%%%%%%%%%%%%%%%%%%%%%%%%%%%%%%%%%%%%%%%%%%%%%%%%%%

%%%%%%%%%%%%% SIMULATION OF DIFFRACTOR %%%%%%%%%%%%%%%%%%%%%%

%%%%%%%%%%%%%%%%%%%%%%%%%%%%%%%%%%%%%%%%%%%%%%%%%%%%%%%%%%%%%%%%%%%%%%%%%%

hologram = normalization_maxmin(hologram, 0, 1);

diffractor = 1 - hologram;

figure

imshow(rot90(diffractor),[]);

% saving image as TIFF image

diffractor = normalization_maxmin(diffractor, 0, 255);

imwrite (rot90(diffractor), gray, 'diffractor.tif','Compression','none');

%%%%%%%%%%%%%%%%%%%%%%%%%%%%%%%%%%%%%%%%%%%%%%%%%%%%%%%%%%%%%

fid = fopen(strcat('diffractor.bin'), 'w');

fwrite(fid, diffractor, 'real*4');

fclose(fid);

%%%%%%%%%%%%%%%%%%%%%%%%%%% END %%%%%%%%%%%%%%%%%%%%%%%%%%%%%%%%%%%%%%%%%%

MATLAB code for propagator simulated by Angular Spectrum Method

%%%%%%%%%%%%%%%%%%%%%%%%%%%%%%%%%%%%%%%%%%%%%%%%%%%%

% PURPOSE: ASM Propagator

%%%%%%%%%%%%%%%%%%%%%%%%%%%%%%%%%%%%%%%%%%%%%%%%%%%%

function [p] = propagator(N, lambda, area, z)

p = zeros(N,N);

for ii = 1:N;

for jj = 1:N

alpha = lambda*(ii - (N/2+1))/area;

beta = lambda*(jj - (N/2+1))/area;

if ((alpha^2 + beta^2) <= 1)

p(ii, jj) = exp(2*pi*i*z*sqrt(1 - alpha^2 - beta^2)/lambda);

end; % if

end

end;

%%%%%%%%%%%%%%%%%%%%%%%%%%%%%%%%%%%%%%%%%%%%%%%%%%%%%%%%%%%%%%

MATLAB code for normalization

%%%%%%%%%%%%%%%%%%%%%%%%%%%%%%%%%%%%%%%%%%%%%%%%%%%%

% PURPOSE: normalization of matrix to given minimal and maximal values

%%%%%%%%%%%%%%%%%%%%%%%%%%%%%%%%%%%%%%%%%%%%%%%%%%%%

%%%% y = k*x + c

%%%% k = (newmax - newmin)/(max0-min0)

%%%% c = newmax - k*max0

%%%%%%%%%%%%%%%%%%%%%%%%%%%%%%%%%%%%%%%%%%%%%%%%%%%%

function [out] = normalization_maxmin(in, newmin, newmax)

[sizey sizex] = size(in);

k = (newmax - newmin)/(max(max(in)) - min(min(in)));

c = newmax - k * max(max(in));

out = k * in + c;

%%%%%%%%%%%%%%%%%%%%%%%%%%%%%%%%%%%%%%%%%%%%%%%%%%%%

MATLAB code for centred Fourier transform

%%%%%%%%%%%%%%%%%%%%%%%%%%%%%%%%%%%%%%%%%%%%%%%%%%%%

% PURPOSE: Centred Fourier transform

%%%%%%%%%%%%%%%%%%%%%%%%%%%%%%%%%%%%%%%%%%%%%%%%%%%%

%%%%%%%%%%%%%%%%%%%%%%%%%%%%%%%%%%%%%%%%%%%%%%%%%%%%

function [out] = FT2Dtat(in)

[Nx Ny] = size(in);

f1 = zeros(Nx, Ny);

for ii = 1:Nx

for jj = 1:Ny

f1(ii, jj) = exp(i*pi*(ii + jj));

end

end

FT = fft2(f1.*in);

out = f1.*FT;

%%%%%%%%%%%%%%%%%%%%%%%%%%%%%%%%%%%%%%%%%%%%%%%%%%%%

MATLAB code for inverse centred Fourier transform

%%%%%%%%%%%%%%%%%%%%%%%%%%%%%%%%%%%%%%%%%%%%%%%%%%%%

% PURPOSE: Centred inverse Fourier transform

%%%%%%%%%%%%%%%%%%%%%%%%%%%%%%%%%%%%%%%%%%%%%%%%%%%%

%%%%%%%%%%%%%%%%%%%%%%%%%%%%%%%%%%%%%%%%%%%%%%%%%%%%

function [out] = FT2Dbacktat(in)

[Nx Ny] = size(in);

f1 = zeros(Nx, Ny);

for ii = 1:Nx

for jj = 1:Ny

f1(ii, jj) = exp(-i*pi*(ii + jj));

end

end

FT = ifft2(f1.*in);

out = f1.*FT;

%%%%%%%%%%%%%%%%%%%%%%%%%%%%%%%%%%%%%%%%%%%%%%%%%%%%
